# Sulfur dioxide inhibits mast cell degranulation by sulphenylation of galectin-9 at cysteine 74

**DOI:** 10.3389/fimmu.2024.1369326

**Published:** 2024-06-17

**Authors:** Jiaru Song, Jie Zheng, Zongmin Li, Ling Fu, Jing Yang, Kun Li, Xiaoqi Yu, Boyang Lv, Junbao Du, Yaqian Huang, Hongfang Jin

**Affiliations:** ^1^ Department of Pediatrics, Peking University First Hospital, Beijing, China; ^2^ State Key Laboratory of Proteomics, Beijing Proteome Research Center, National Center for Protein Science Beijing, Beijing Institute of Lifeomics, Beijing, China; ^3^ Key Laboratory of Green Chemistry and Technology, Ministry of Education, College of Chemistry, Sichuan University, Chengdu, China; ^4^ State Key Laboratory of Vascular Homeostasis and Remodeling, Peking University, Beijing, China

**Keywords:** endogenous sulfur dioxide, mast cell, degranulation, galectin-9, sulphenylation

## Abstract

**Objectives:**

Mast cell (MC) degranulation is a key process in allergic reactions and inflammatory responses. Aspartate aminotransferase 1 (AAT1)-derived endogenous sulfur dioxide (SO_2_) is an important regulator of MC function. However, the mechanism underlying its role in MC degranulation remains unclear. This study aimed to investigate the mechanism by which endogenous SO_2_ controlled MC degranulation.

**Methods:**

HMC-1 and Rat basophilic leukemia cell MC line (RBL-2H3) were used in the cell experiments. SO_2_ content was detected by *in situ* fluorescent probe. MC degranulation represented by the release rate of MC β-hexosaminidase was determined using a colorimetric assay. Sulfenylation of galectin-9 (Gal-9) in MCs and purified protein was detected using a biotin switch assay. Liquid chromatography-tandem mass spectrometry (LC-MS/MS) was used to determine the exact sulfenylation sites of Gal-9 by SO_2_. Animal models of passive cutaneous anaphylaxis (PCA) and hypoxia-driven pulmonary vascular remodeling were used to investigate the effect of SO_2_ on mast cell activation *in vivo*. Site-directed mutation of Gal-9 was conducted to confirm the exact site of SO_2_ and support the significance of SO_2_/Gal-9 signal axis in the regulation of MC degranulation.

**Results:**

Degranulation was increased in AAT1-knockdowned MCs, and SO_2_ supplementation reversed the increase in MC degranulation. Furthermore, deficiency of endogenous SO_2_ contributed to IgE-mediated degranulation *in vitro.* Besides, SO_2_ inhibited IgE-mediated and hypoxia-driven MC degranulation *in vivo*. Mechanistically, LC-MS/MS analysis and site-directed mutation results showed that SO_2_ sulfenylated Gal-9 at cysteine 74. Sulfenylation of the 74^th^ cysteine of Gal-9 protein was required in the SO_2_-inhibited MC degranulation under both physiological and pathophysiological conditions.

**Conclusion:**

These findings elucidated that SO_2_ inhibited MC degranulation via sulfenylating Gal-9 under both physiological and pathophysiological conditions, which might provide a novel treatment approach for MC activation-related diseases.

## Introduction

As an important type of innate immune cells, the mast cell (MC) is widely distributed throughout the body and is often in contact with the external environment through the skin, respiratory tract, and intestinal tract (1). MCs are sentinel cells that protect the host from germs and parasites and are essential for innate immunity (1). When MCs are stimulated by exogenous or endogenous stimuli, they can quickly release the preformed particles from the cytoplasm to extracellular environment (2). MC granules mainly consist of proteoglycans, protein enzymes, biogenic amines, lysosomal enzymes, cytokines, and growth factors (3). It has been shown that MC trypsin or chymase can produce an inflammatory response (4, 5). MCs may directly contribute to vascular remodeling through increased matrix metalloproteinase (MMP) activity (6). MC degranulation generally occurs via two pathways: the classical IgE-mediated pathway and non-IgE-mediated pathway. The former mediates MC degranulation by binding the high-affinity Fc receptor for IgE (FcϵRI) of MCs (7), while the latter induces MC degranulation by exposure to stem cell factors, endothelin-1, and neuropeptides, etc (8–10). Collectively, MC degranulation is a crucial pathological process of mast cell activation-related disease including allergic situations, allergic dermatitis, tumor angiogenesis and allergic airway inflammation (11–14). Correspondingly, the inhibitors of MC degranulation are developed and used in the clinical treatment of many diseases (15–17). However, the mechanism underlying MC degranulation has not yet been fully elucidated. Therefore, identifying novel controller of MC degranulation remains a key scientific item.

Sulfur dioxide (SO_2_) is a newly discovered gaseous signaling molecule. Endogenous SO_2_ is generated from a reaction catalyzed by aspartate aminotransferase (AAT), using L-cysteine as the substrate. Endogenous SO_2_/AAT pathway exists in vascular endothelial cells, adipocytes, cardiomyocytes, alveolar epithelial cells, and fibroblasts (18–22). Previous studies showed that overexpression of AAT1 inhibited the secretion of tumor necrosis factor-α (TNF-α)-generated inflammatory factors in 3T3-L1 adipocytes, such as monocyte chemoattractant protein-1 (MCP-1) and interleukin (IL)-8 (23). In colon tissue, SO_2_ supplementation attenuated the mRNA expression of TNF-α, IL-1β, and IL-6 in a 2,4,6-trinitrobenzenesulfonic acid-induced colitis model (24). In a rat sepsis model generated via cecal ligation and puncture (CLP), SO_2_ supplementation significantly inhibited the CLP-induced expression of Toll-like receptors-4 and pyrin domain-containing protein 3 (NLRP3) in rat myocardial tissues, and SO_2_ exerted anti-inflammatory effects through the NLRP3 inflammasome signaling pathway, thereby attenuating sepsis-induced cardiac dysfunction (25). Those abovementioned studies indicate that endogenous SO_2_ is involved in the inflammatory response. Considering that Zhang et al. have identified an endogenous SO_2_/AAT1 system in MCs (26), we propose that SO_2_ might be crucial for the regulation of MC-related processes or degranulation.

It is reported that SO_2_ switches the function of target proteins through sulfenylation at thiol of cysteine residue and exerts various biological effects (27–29). For example, SO_2_ inhibited inflammatory response through sulfenylation of nuclear factor κB (NF-κB) p65 at cysteine (Cys) 38 in acute lung damage models in rats (29). SO_2_ upregulated the sulfenylation of mothers against decapentaplegic homolog 3 (Smad3) and inhibited the activation of downstream transforming growth factor β (TGF-β) signaling pathway (30). However, it remains unclear whether SO_2_ regulates MC degranulation via sulfenylation of certain target protein.

Therefore, in the present study, we aimed to exploreed the regulatory effect of SO_2_ on MC degranulation both *in vivo* and *in vitro*. Furthermore, the chemical modification mechanism by which SO_2_ controlled MC degranulation was investigated using the cell and cell-free experiments.

## Materials and methods

### Cell culture and treatment

The human mast cell line (HMC-1) was purchased from China Infrastructure of Cell Line Resources Center. Iscove’s Modified Dulbecco’s Medium (IMDM) containing 1% penicillin and streptomycin, 10% fetal bovine serum, and 2 mM L-glutamine was utilized to cultivate HMC-1 cells in an incubator with 5% CO_2_ at 37°C. Hypoxic conditions were maintained at 1% O_2_ concentration using a hypoxic chamber (Biospherix C21, USA). Freshly prepared 100 μM Na_2_SO_3_/NaHSO_3_ (Sigma, USA) was used as the SO_2_ donor. The working concentration of C48/80 (MCE, China) was 20 μg/ml. The rat basophilic leukemia cell MC line (RBL-2H3) was purchased from China center for culture collection and cultured in MEM medium with 10% FBS. RBL-2H3 cells were incubated overnight with 100 ng/ml anti-DNP-IgE (Sigma-Aldrich, USA) at 37°C. The following day, cells were washed and stimulated with 100 ng/ml DNP-HSA (Biosearch, USA) for 1 hour at 37°C.

### Lentivirus transfection

HMC-1 cells were infected with lentivirus containing the small hairpin RNA (shRNA) AAT1 (Cyagen, China) to construct AAT1-knockdown MCs. Briefly, the lentivirus containing AAT1-shRNA was added when the cell density was between 60% and 70%, with a multiplicity of infection of 10 in compliance with the manufacturer’s guidelines. Fresh complete media were added after 24 h, and kept at 37°C for the entire night. For a week, puromycin (4 μg/ml) was used for screening the infected cells to get stable AAT1-shRNA-infected MCs. HMC-1 cells infected with lentivirus carrying scrambled shRNA were used as a control according to the same protocol.

### *In situ* detection of endogenous SO_2_ levels in cells by fluorescent probe

SS-1 is a chemo-selective fluorescent probe for *in situ* detection of SO_2_, kindly provided by Prof. Xiaoqi Yu and Prof. Kun Li from Sichuan University (31). Briefly, cells were incubated with SO_2_ fluorescent probe for half an hour, followed by three PBS rinses and a minimum of ten minutes of fixation with 4% paraformaldehyde. After rinsing the cells as previously mentioned, the proper concentration of DAPI was applied to allow nuclear to be seen. Green fluorescence observed using a confocal microscope (Zeiss, LSM900) indicated the presence of SO_2_. The signal intensity of fluorescence was measured using Image J (NIH, USA) software.

### Determination of AAT activity by colorimetric assay

RBL-2H3 cells were collected using PBS and sonicated to obtain the cell lysate. Ear tissue was homogenized with PBS using a tissue homogenizer, centrifuged, and the supernatant was collected. AAT activity of cells and ear tissue was measured according to the instructions of AAT activity assay kit (Nanjing Jiancheng, Nanjing, China). Briefly, the substrate solution and test samples were added into a 96-well plate, and incubated at 37°C for 30 minutes. Then, 2,4-dinitrophenylhydrazine was added to each well and the plate was placed at 37°C for 20 minutes. Afterward, sodium hydroxide solution (200μl, 0.4 M) was added and gently mixed into each well. The plate was incubated at room temperature for 15 minutes. Finally, the optical density was measured at the absorption wavelength of 510 nm. Sodium pyruvate standard solution (2 μM) was used as the standard substance, and diluted according to the instructions to obtain different concentrations. The absolute absorbance values for each concentration were plotted as the y-axis, and the corresponding absorbance units were plotted as the x-axis to create a standard curve. AAT activity was calculated from the standard curve after colorimetric determination. The AAT activity of cells and ear tissue was corrected for homogenate protein concentration.

### The release rate of β-hexosaminidase by using colorimetric assay

A β-N-acetylglucosaminase kit was used to evaluate the release rate of β-hexosaminidase from MCs (Yuanmu, China). After isolating the cells by centrifugation, the cell culture supernatant and MCs were collected for examination. MCs were sonicated and centrifuged to obtain cell solution. A mixture containing 10 µl of sample (cell culture supernatant or cell solution) and 50 µl of substrate buffer was incubated at 37°C for 15 minutes. Then the reaction was stopped by adding alkaline solution and the absorbance value was measured at 400 nm. Each liter of the sample was acted with the substrate at 37°C for 1 min and hydrolyzed to produce 1 μmol of p-nitrophenol as 1 unit of enzyme activity.

### Bioinformatics analysis

Venn analysis is an analysis of different datasets used to categorize the intersecting data. Three datasets were used in this study: vascular smooth muscular cell (VSMC) sulfenylome dataset (30), positive regulation of MC activation Gene Ontology dataset (GO: 0033005), and negative regulation of MC activation Gene Ontology dataset (GO: 0033004). In this study, the number of shared genes was statistically analyzed using the sulfenylome and positively regulated/negatively regulated MC activation datasets to show the similarity and overlap of gene compositions between the different datasets.

### Detection of sulfenylation of Galectin-9 by biotin switch assay

The sulfenylation of Gal-9 in HMC-1 cells and human purified Gal-9 protein (MCE, China) were quantified using BSA (30). Briefly, HMC-1 cells were lysed in non-denatured lysis buffer (Applygen, China) supplemented with 5 mM DAz-2 for 20 minutes. The supernatant was gathered by a centrifugation at 4°C at a speed of 16000 g for 5 minutes, and then incubated at 37°C with gentle shaking for 2.5 h. Subsequently, 250 μM p-biotin (Cayman, USA) was added, and the mixture was incubated for two hours at 37°C. UltraLink™ Immobiolized NeutrAvidin ™ (Thermo Fisher Science, USA) was added at a 1:10 volume ratio and incubated at 4°C for 4 h. The beads were washed 5 times with PBS and centrifuged to enrich sulfenylated protein. Finally, non-denatured loading buffer was added and heated for ten minutes at 100°C. Western blot was conducted to quantify the level of Gal-9 sulfenylation.

Human purified Gal-9 protein was divided into three groups: control, SO_2_ and SO_2_+Dithiothreitol (DTT) groups. Each sample used 0.2 μg of protein. The protein was incubated with SO_2_ and DTT at 37°C for 2 h. The working concentration of SO_2_ and DTT were both 100 μM. After the incubation, the sulfenylation of Gal-9 was detected according to the same protocol described in the cell experiments.

### Sequence homology analysis of Gal-9 across different species

From the UniProt database (https://www.uniprot.org/), the Gal-9 protein sequence for each species was obtained. The protein IDs were listed as follows: human (O00182), mouse (O08573), rat (P97840), bovine (Q3MHZ8), Camelus dromedarius (A0A5N4D4M7), and pig (Q9XSM9). BioEdit software was used for the homology analysis of the above sequences.

### Detection of sulfenylation of Gal-9 by liquid chromatography-tandem mass spectrometry

Human purified Gal-9 protein was dissolved in PBS (10 μg in 30 μl). Then 100 μM SO_2_ and 10 mM dimethyl ketone were added, shaken gently, and incubated for 2 hours at 37°C. The protein samples were loaded into a non-reducing loading buffer and separated using 12% sodium dodecyl sulfate-polyacrylamide gel electrophoresis (SDS-PAGE). The coomassie bright blue R-250 dye was applied to the protein gel, then enzymatically cut. LC-MS/MS was utilized for the extraction and identification of the peptide mixture. The pFind 3 software, developed by Professor He Simin and the members of his group at the Institute of Computing Technology, Chinese Academy of Sciences, was used to analyze the mass spectra (32).

### Plasmid electrotransformation

HMC-1 and RBL-2H3 cells were transfected with plasmids using the SF 4D-NucleofectorTM X Solution Kit (Lonza, Germany). Electrotransformation mix (100 μl) containing 1 μg of plasmid was prepared in compliance with the manufacturer’s guidelines. The pcDNA3.1 vectors for Gal-9-WT-His, Gal-9-C74S-His and Gal-9-C312S-His were constructed (General, Anhui, China). Briefly, the cells underwent transfection and were left to stand at room temperature for ten minutes. Then cells were gently resuspended, aspirated into culture wells, and gently mixed with a pre-warmed medium. After six hours, the medium was replaced with a new one.

### Western blot

Following the lysis of the cells in RIPA buffer, the protein concentrations were determined using a BCA kit (Beyotime, China). Proteins in equal amounts were separated using 12% SDS-PAGE and then transferred onto nitrocellulose membranes. These membranes were then blocked using a blocking solution that contained 5% milk powder. The primary antibodies were incubated with the following dilutions: anti-AAT1 (1:1000; Abcam, USA), anti-β-tubulin (1:2000; Zsbio, China), anti-Galectin-9 (1:1000; Proteintech, China), and anti-His (1:1000; Zsbio, China). The corresponding secondary antibodies were used subsequently. The bands incubated with chemiluminescent detection reagents were analyzed by a FluorChem M MultiFluor system (Protein Simple, USA).

### Animal model

Mice were purchased from Si Pei Fu Biotechnology Co., Ltd (Beijing, China) and housed in the Experimental Animal Center of Peking University First Hospital. Mice were housed in a specific pathogen-free and viral antibody-free animal facility in accordance with the guidelines established by the Institutional Committee on Animal Use and Care (IACUC) and Laboratory Animal Resource Center (LARC).

For IgE-mediated passive cutaneous anaphylaxis (PCA) model, the animal study was approved by the Animal Research Ethics Committee of Peking University First Hospital (Ethics No.: J2024039). Briefly, the mouse PCA model was constructed by an intradermal injection with 500 ng of anti-DNP-IgE in 20 μl PBS in the left ear and an intravenously challenge with DNP-HSA (50 μg in saline containing 1% Evans blue) after 24h in the eight-week-old female BALB/c mice. Thirty minutes after the challenge, skin areas were photographed and the thickness of the ears was measured, after which the mice were euthanized. Evans blue dye was extracted by incubating the skin tissues in 300 μl of formamide for 24 h at 63°C, after which absorbance was measured on a spectrophotometer at 620 nm. Mice in IgE+SO_2_ group were intraperitoneally injected three days with SO_2_ donor (Na_2_SO_3_:68.04 mg/kg; NaHSO_3_:18.72 mg/kg body weight) before IgE sensitization (33). Mice in control group received three intraperitoneal injections with the same amount of physiological saline and an intradermal injection with 20 μl of PBS. SO_2_ donor was freshly dissolved in saline.

The animal study about chronic hypoxia/SU5416 (SuHx)-stimulated mouse model was approved by the Animal Research Ethics Committee of Peking University First Hospital (Ethics No.: J2023008). C57BL/6 mice were exposed to normoxia (ambient air, 21% O_2_) or hypoxia (10% O_2_) in a ventilated chamber (Ox-100, TOW, Shanghai, China) for 3 weeks. The mice in the SuHx and SuHx+SO_2_ group received a single weekly subcutaneous injection of the VEGFR2 antagonist SU5416 at 20mg/kg, while the normoxia group received a vehicle injection (34). SO_2_ donors (Na_2_SO_3_:68.04 mg/kg; NaHSO_3_:18.72 mg/kg body weight) were administered intraperitoneally daily.

### Determination of SO_2_ content by high-performance liquid chromatography with a fluorescence detector

SO_2_ levels in ear tissues were measured by HPLC-FD (Agilent, Palo Alto, CA, USA) as described in the previous study (30). In brief, ear tissue was homogenized with PBS using a tissue lyser, centrifuged, and the supernatant was collected. The SO_2_ in the sample was reduced to sulfhydryl compounds by sodium borohydride, and then labeled with monobromobimane to generate fluorescent derivative. Perchloric acid was used to deproteinize. After centrifugation, the supernatant was neutralized by Tris-HCl (pH 3.0) and prepared for HPLC-FD analysis. The fluorescent derivatives were separated by chromatographic column and detected at excitation/emission wavelength of 392/479 nm.

### Toluidine blue staining for *in vivo* detection of mast cell degranulation

Mouse tissues were fixed with 10% formalin and embedded in paraffin. The fixed tissues were cut into 4 µm sections. Sections were dewaxed and incubated with toluidine blue dye solution (Solarbio, China) for 10 min. Excess dye was then washed away and the background color was separated with 95% alcohol for 5min. The quantification of mast cells and their degranulation status relied on the identification of abnormal morphology and the presence of extracellular cytoplasmic granules under a light microscope using 400× magnification (Olympus, Japan) (35). The number of degranulated mast cells was counted in two fields per section.

### Hematoxylin-eosin staining

HE staining was conducted following standard procedures. Briefly, tissue sections were immersed in hematoxylin solution, followed by rinsing in PBS to eliminate excess stain. Subsequently, the sections were differentiated in 1% acid alcohol for 1 s, and rinsed in water for 1 min. Finally, sections were sequentially stained with eosin dye, dehydrated in gradient ethanol and xylene, and mounted with resinene.

### Elastic-Van Gieson staining for arterioles in mouse lung tissues

The sections were soaked in xylene to dewax, and then rehydrated in gradient ethanol. Subsequently, the sections were stained with the Verhoeff staining solution (a mixture of alcohol hematoxylin, ferric chloride, and iodine solution) for 30 min. Following this, the sections were stained with Van Gieson’s solution (a mixture of saturated picric acid and acidic magenta in a volume ratio of 9:1) for 1–3 minutes, followed by a quick water wash. Finally, the sections were sequentially immersed in anhydrous ethanol and xylene for 1–5 minutes, and sealed with neutral resin. Images were acquired and analyzed using Leica Q550 CW. Total vessel area was defined as the area within the lamina elastica externa, and lumen area was defined as the area within the lamina elastica interna. Medial area is defined as the area between the lamina elastica externa and lamina elastica interna. The media area was expressed as a percentage of the total external area of the vessel (36, 37). The vessel median thickness was measured at four points at 12, 3, 6 and 9 o’clock on each vessel section, and the average value was calculated (38).

### Statistical analysis

For statistical analysis, Graphpad Prism 8 and SPSS17.0 were both utilized, and the results were shown as mean ± SD. GraphPad Prism 8 software was used to generate graphs. The two-tailed Student’s *t*-test was performed for the comparison between two groups, and one-way ANOVA followed by Bonferroni post-doc analysis was used to compare the difference among multiple groups. A significance level of P < 0.05 was applied.

## Results

### Endogenous SO_2_ inhibited the degranulation of MCs under physiological condition

**Figure 1:**
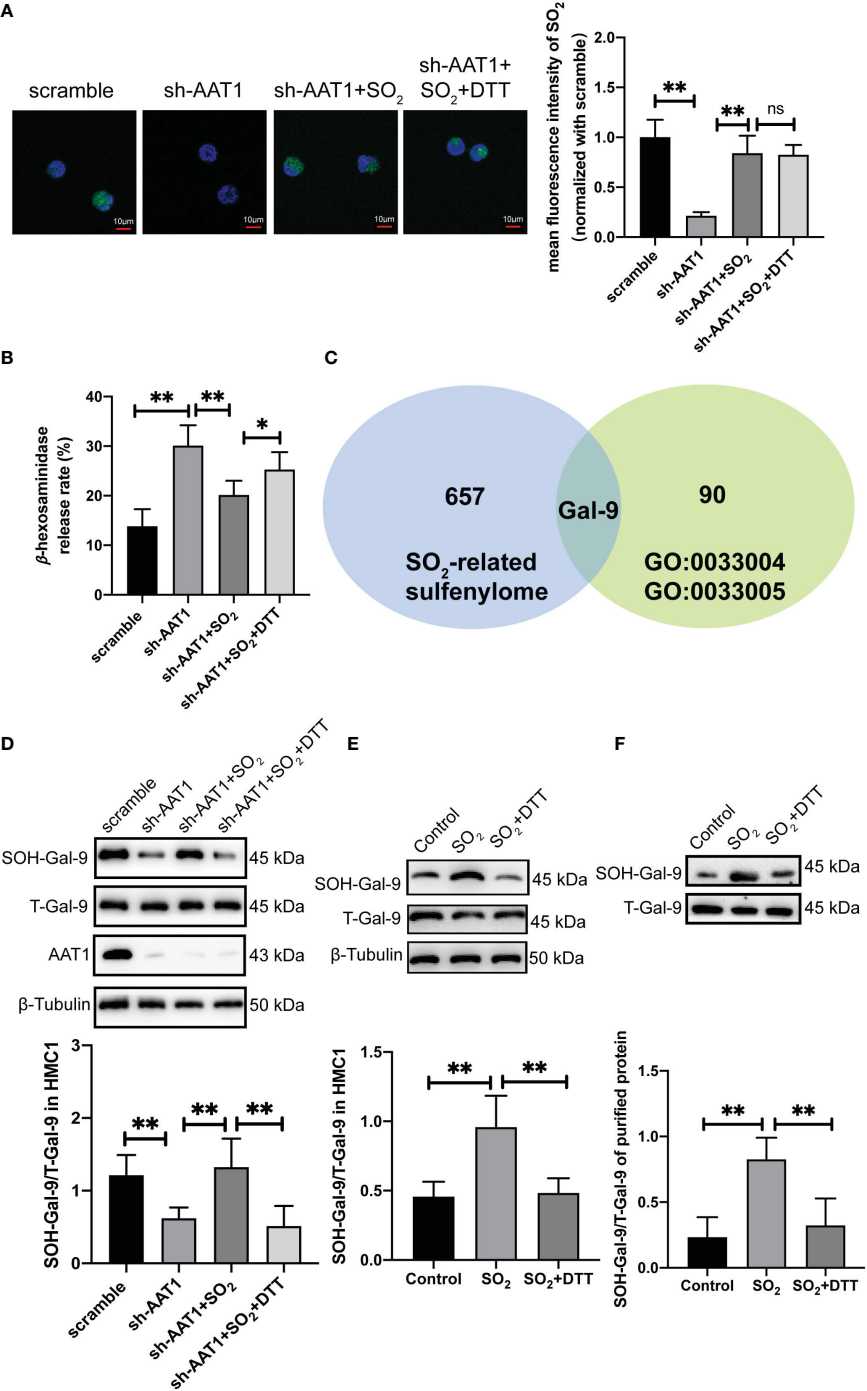
Endogenous SO_2_ increased sulfenylation of Gal-9. AAT1-knockdowned HMC-1 cells supplemented with SO_2_ donor (100 μM) or SO_2_ donor combined with DTT (100 μM) for 24h. **(A)** SO_2_ production in HMC-1 cells was tested with *in situ* fluorescent SO_2_ probe (green color, scale bar: 10 μm) (n=9). **(B)** Using a colorimetric assay, the rate of β-hexosaminidase release from mast cells was measured (n=9). **(C)** Venn diagram of gene composition in sulfenylome (blue) and Gene Ontology datasets (green). **(D)** BSA method was used to determine Gal-9 sulfenylation in the cells of scramble, sh-AAT1, sh-AAT1+SO_2,_ and sh-AAT1+SO_2_+DTT groups (n=9). **(E)** BSA method was used to determine Gal-9 sulfenylation in the cells of control, SO_2,_ and SO_2_+DTT groups (n=9). **(F)** BSA method was used to determine Gal-9 sulfenylation in the purified protein (n=9). Data were expressed as mean ± SD. A one-way ANOVA with *post hoc* Bonferroni was employed in the statistical analysis. *P<0.05, **P<0.01, ns, not significant.

MC degranulation activation is a crucial initiation and development process of the immune inflammatory response. The β-hexosaminidase release is a landmark event of MC degranulation. It has been confirmed that AAT1 catalyzes the generation of endogenous SO_2_ in MCs (26). In the present study, we verified the effect of endogenous SO_2_ on MC degranulation under physiological condition by employing AAT1 knockdown and SO_2_ rescue. The data showed that AAT activity and SO_2_ content were decreased in AAT1-knockdowned HMC-1 compared with scrambled cells, while the supplementation of SO_2_ donor restored the SO_2_ content in cells of sh-AAT1 group (**Figure 1A**, **Supplementary Figure 1A**). Simultaneously, the release of β-hexosaminidase in HMC-1 cells was significantly increased after AAT1 knockdown compared with that in the scramble group, which was reversed by SO_2_ donor (**Figure 1B**). However, in the MCs transfected with scramble lentivirus, SO_2_ donor increased the SO_2_ content in the cells but did not affect MC degranulation (**Supplementary Figure 2**). The above findings suggested that AAT1-derived endogenous SO_2_ might be an important stabilizer by suppressing MC degranulation under physiological condition.

### SO_2_ increased sulfenylation of Gal-9

Subsequently, we explored the probable mechanism underlying endogenous SO_2_-controlled MC degranulation. As shown in **Figure 1B**, a thiol-reducing agent DTT significantly abolished the SO_2_-suppressed β-hexosaminidase release, suggesting that a thiol-dependent chemical modification might be involved in the regulation of SO_2_-controlled MC degranulation. Furthermore, we screened the candidate target proteins by SO_2_ from a SO_2_-mediated sulfenylome dataset as previously described (30). Huang et al. found that 1137 thiol groups of cysteine residue on 658 proteins were responsive to SO_2_ in VSMC (30). Based on the GO dataset, 16 genes were related to negative regulation for MC activation and 75 genes were related to positive regulation. Venn analysis was performed on the SO_2_ sulfenylome dataset using positively and negatively regulated MC activation datasets. The only candidate gene, LGALS9, encoding the protein Gal-9, was found to regulate MC activation, and the thiol group of the Gal-9 protein could be modified by SO_2_-mediated sulfenylation (**Figure 1C**).

Based on the grouping shown in **Figure 1B**, we verified whether endogenous SO_2_ sulfenylated the Gal-9 protein in HMC-1 cells. BSA results showed that the sulfenylation of Gal-9 protein in the MCs of the sh-AAT1 group was substantially lower than that of the scramble group. Compared with the sh-AAT1 group, Gal-9 sulfenylation increased after SO_2_ supplementation, while the sulfenylation of Gal-9 by SO_2_ was significantly decreased when the cells treated with DTT supplementation (**Figure 1D**). These results indicated that HMC-1 cell-derived SO_2_ modified the thiol group of the Gal-9 protein by sulfenylation.

Furthermore, an enhanced sulfenylation of Gal-9 in HMC-1 cells of SO_2_ group was shown compared with the control group, which was reversed by the treatment of DTT (**Figure 1E**). Subsequently, the SO_2_-induced Gal-9 sulfenylation was verified in the cell-free experiment. The BSA results showed that the SO_2_ treatment upregulated the sulfenylation of Gal-9 in the purified human Gal-9 protein, while DTT supplementation blocked SO_2_-induced Gal-9 sulphenylation (**Figure 1F**). These findings suggested that Gal-9 was sulfenylated by SO_2_ in cellular and cell-free experiments.

### Sulfenylation of Gal-9 protein occurred at Cysteine74

**Figure 2:**
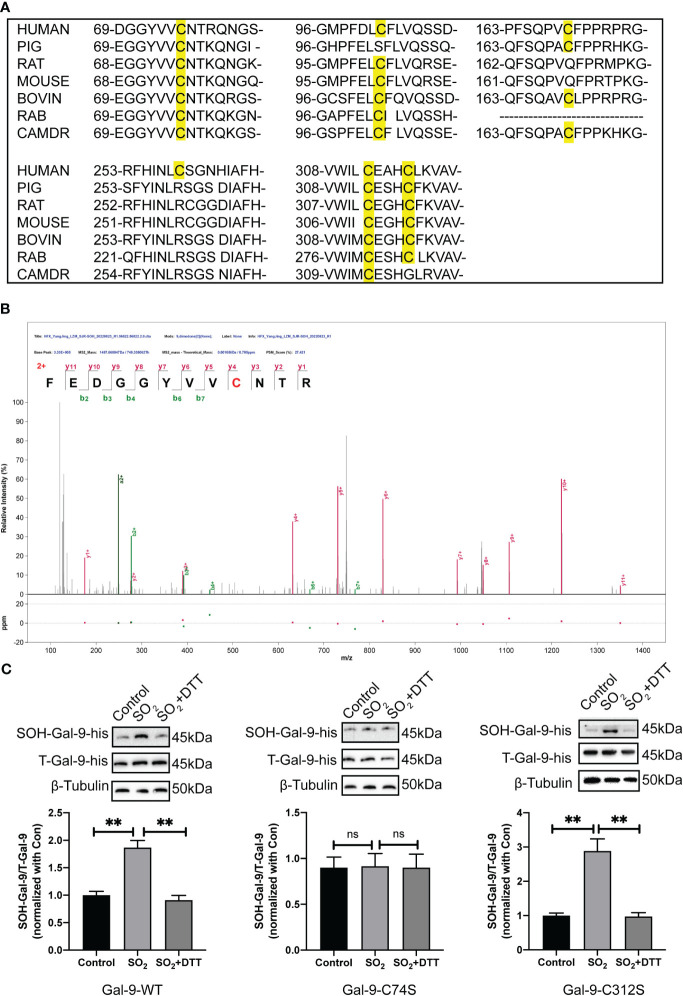
The sulfenylation of Gal-9 protein occurred at Cysteine 74. **(A)** Sequence conservation comparison of Gal-9 between different species. **(B)** The LC-MS/MS approach was applied to verify the sulfenylated site of Gal-9 purified protein by SO_2._ The discovery of a strand of y and b ions in the MS/MS spectra identified their sequences definitively as FEDGGYVVC*NTR, in which C* is a dimedone-modified residue. **(C)** BSA method was used to determine Gal-9 sulfenylation in HMC-1 transfected with Gal-9-WT, Gal-9-C74S and Gal-9-C312S plasmids (n=9). Data were expressed as mean ± SD. A one-way ANOVA with *post hoc* Bonferroni was employed in the statistical analysis. **P<0.01, ns, not significant.

To further investigate the exact sulfenlyation site on the Gal-9, a bioinformatics analysis, LC-MS/MS, and site-directed mutation were used. Firstly, as showed in the **Figure 2A**, human Gal-9 has six cysteine residues: Cys74, Cys102, Cys169, Cys259, Cys312, and Cys316. Homologous conservation analysis of cysteine sites was performed on the Gal-9 sequences of different species, and it was found that the cysteine residues 74 and 312 in humans, mice, rats, cows, pigs, and other species were highly conserved (**Figure 2A**).

Secondly, LC-MS/MS results showed that sulfenylation of purified Gal-9 protein by SO_2_ occurs at the residue of cysteine 74 (**Figure 2B**), which was in accordance with the finding demonstrated in the SO_2_-mediated sulfenylome in the previous study. Next, we constructed Gal-9-wild type (WT), Gal-9-C74S (mutation of cysteine-74 to serine), and Gal-9-C312S (mutation of cysteine-312 to serine) mutant plasmids. HMC-1 cells were transfected using the electrotransfer method. Here, Gal-9-C312S mutant plasmid was used as negative control. The results of BSA detection suggested that SO_2_ treatment promoted the sulfenylation of Gal-9 in cells transfected with Gal-9-WT and Gal-9-C312S mutant plasmids. However, SO_2_-induced Gal-9 sulfenylation was not found in cells transfected with Gal-9-C74S mutant plasmid (**Figure 2C**). These above findings suggested that sulfenylation of Gal-9 by SO_2_ occurred at the 74^th^ cysteine residue.

### Deficiency of endogenous SO_2_ contributed to IgE-mediated degranulation *in vitro*

**Figure 3:**
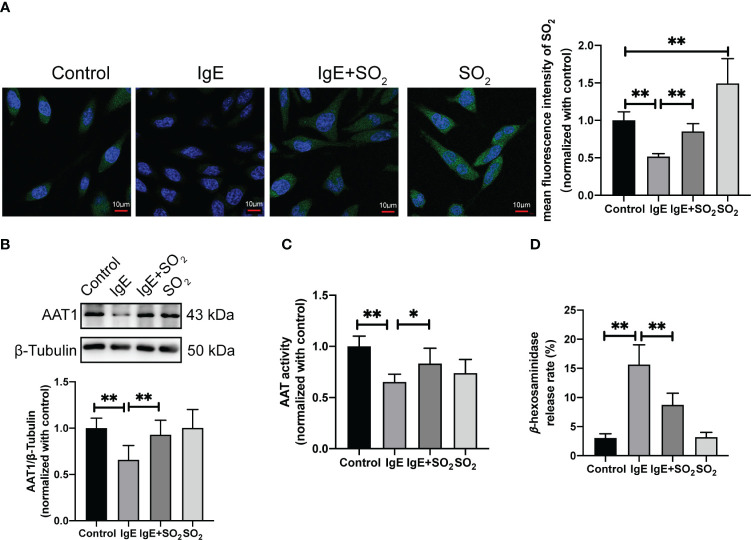
Impact of endogenous SO_2_ on IgE-mediated degranulation in RBL-2H3 cells. RBL-2H3 cells were incubated overnight with anti-DNP-IgE (100 ng/ml) and stimulated with DNP-HSA (100 ng/ml) for 1h. SO_2_ donor (100 μM) was added 1 hour before anti-DNP-IgE and DNP-HSA stimulation. **(A)** SO_2_ production in RBL-2H3 cells was tested with *in situ* fluorescent SO_2_ probe (green color, scale bar: 10 μm) (n = 9). **(B)** AAT1 expression in RBL-2H3 cells was measured by western blot (n = 9). **(C)** AAT activity in RBL-2H3 cells was detected by colorimetric assay (n = 9). **(D)** The release rate of β-hexosaminase in RBL-2H3 cells was determined by using colorimetric assay (n = 9). Data were expressed as mean ± SD. A one-way ANOVA with *post hoc* Bonferroni was employed in the statistical analysis. *P<0.05, **P<0.01.

Considering IgE is a classical stimulator of MC degranulation, we further investigated the effect of SO_2_ on MC degranulation in the IgE-challenged MCs and mouse models. The rat basophilic leukemia cells, RBL-2H3, are a tumor analogue of mast cells with a high expression of FcϵRI on the cell surface, and can be activated by the IgE-antigen complex (39, 40). Our results showed a significant reduction in SO_2_ content, AAT1 protein expression, and AAT activity within IgE-insulted RBL-2H3 cells compared with the control group (**Figures 3A–C**). Concurrently, MC degranulation was increased in IgE-treated cells compared with IgE-untreated cells (**Figure 3D**). Also, the supplementation of SO_2_ donor restored the SO_2_ content and reduced MC degranulation in IgE-stimulated cells (**Figures 3A, D**). Those *in vitro* findings suggested that IgE stimulation downregulated the endogenous SO_2_/AAT1 pathway in MC, as well as the deficiency of endogenous SO_2_ might participate in IgE-induced MC degranulation.

### SO_2_ inhibited IgE-mediated MC activation in passive cutaneous anaphylaxis mouse

**Figure 4:**
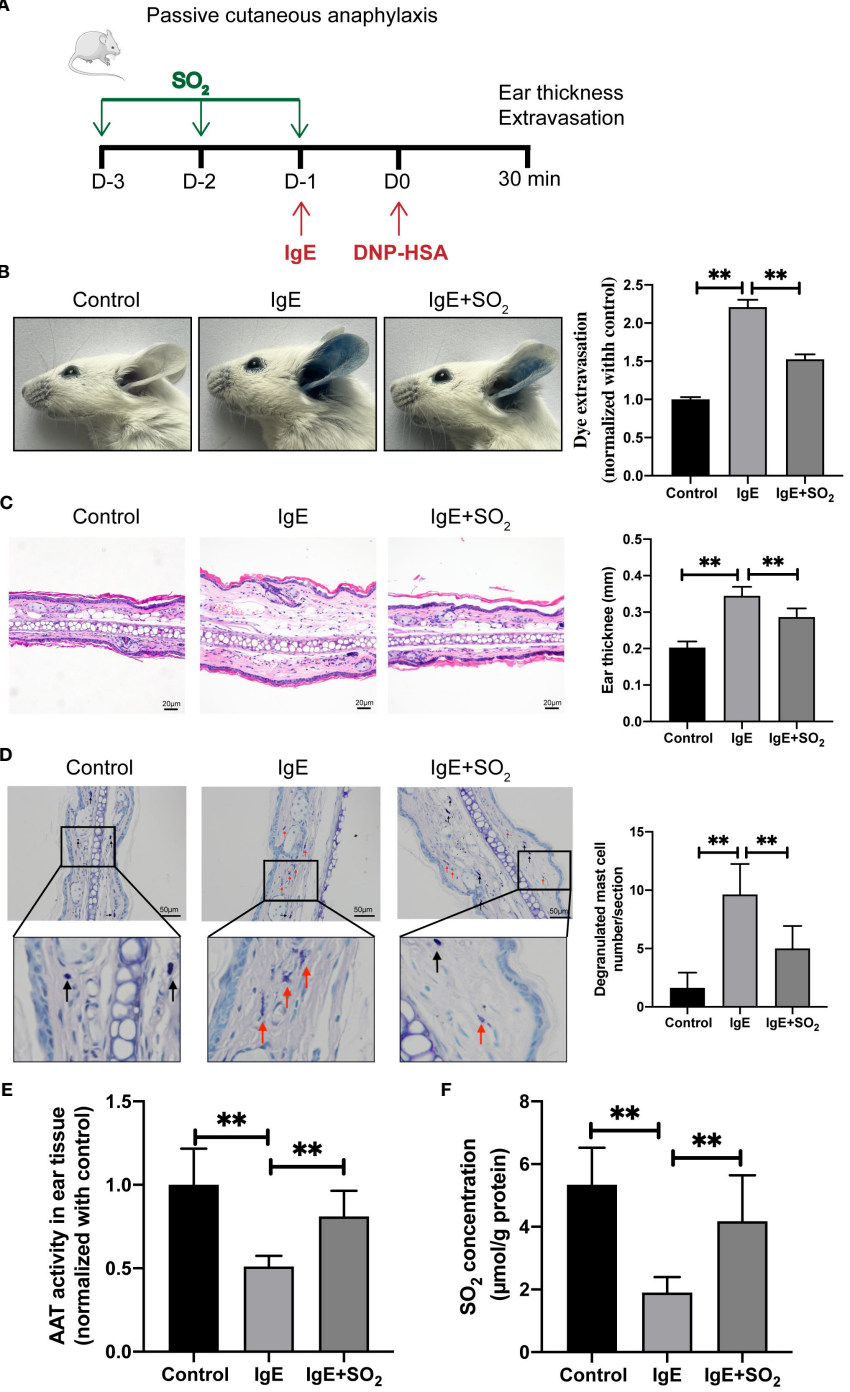
SO_2_ supplementation suppressed MC activation in passive cutaneous anaphylaxis mouse. **(A)** Schematic diagram for the IgE/HSA-induced PCA mouse model and mouse grouping. BALB/c mice were sensitized with 500 ng anti-DNP-IgE and challenged with 50 μg DNP-HSA with or without intraperitoneal pretreatment of SO_2_ donor (Na_2_SO_3_: 68.04 mg/kg; NaHSO_3_: 18.72 mg/kg body weight). **(B)** Representative ear images and quantitative analysis of Evans blue dye extravasation of mouse ears (n = 8). **(C)** Representative HE staining of ear sections. Scale bar: 20 μm. Ear swelling calculated by ear thickness (n = 8). **(D)** Representative toluidine blue staining of ear sections. Scale bar: 50 μm. Degranulated mast cells in ear skin section were counted (n = 8). Black and red arrows indicate non-degranulated and degranulated mast cells, respectively. **(E)** AAT activity in ear tissue was detected by colorimetric assay (n = 8). **(F)** SO_2_ contents in the mouse ear tissues were detected by HPLC-FD (n = 8). Data were expressed as mean ± SD. A one-way ANOVA with *post hoc* Bonferroni was employed in the statistical analysis. **P<0.01.

Furthermore, we constructed a mouse IgE-mediated PCA model to investigate the effect of SO_2_ on the mast cell-dependent inflammation *in vivo* (**Figure 4A**). Local anti-DNP-IgE sensitization and systemic administration of DNP-HSA together with Evans blue triggered a remarkable dye extravasation and ear swelling, but reduced AAT activity and SO_2_ content in the IgE-sensitized ear compared with the control group (**Figure 4**). As observed in the *in vitro* experiments, the pretreatment of SO_2_ donor recovered the SO_2_ content in ear tissue, and subsequently prevented the dye extravasation and ear swelling in the IgE-challenged mouse (**Figure 4**), further reinforcing that SO_2_ suppressed IgE-related MC activation and inflammation.

### SO_2_ restrained hypoxia-driven lung MC degranulation and pulmonary vascular remodeling

**Figure 5:**
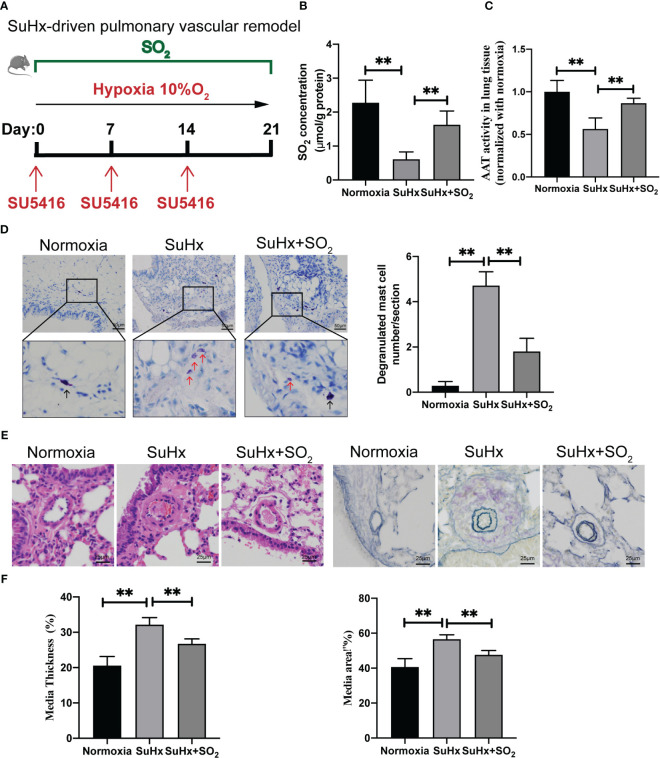
SO_2_ supplementation inhibited hypoxia-driven lung MC degranulation and pulmonary vascular remodeling. **(A)** Schematic diagram for the hypoxia-driven pulmonary vascular remodeling mouse model and mouse grouping. C57BL/6J mice were exposed to hypoxia (10% O_2_) combined with SU5416 for 3 weeks with or without intraperitoneal injections of the SO_2_ donor (Na_2_SO_3_:68.04 mg/kg; NaHSO_3_:18.72 mg/kg body weight) administered daily. **(B)** SO_2_ contents in the mouse lung tissues were detected by HPLC-FD (n = 5~7). **(C)** AAT activity in lung tissues was detected by colorimetric assay (n = 5~7). **(D)** Representative toluidine blue staining of lung sections. Scale bar: 50 μm, Black and red arrows indicate non-degranulated and degranulated mast cells. Degranulated mast cells in lung sections were counted (n = 5~7). **(E)** Representative HE staining of lung sections (n = 5~7). Scale bar: 25 μm. Verhoeff’s-Van-Gieson staining of arterioles in lung tissues (n = 5~7). Scale bar: 25 μm. **(F)** Quantification of the percentage of pulmonary small artery media thickness and area based on the Elastic-Van Gieson staining. The percentage of media thickness is defined as [(2 × medial wall thickness/external diameter) × 100]. The percentage of media area is defined as a ratio of the medial vascular wall area to the total vessel area. 38–56 blood vessels were counted in each group of mice. Data were expressed as mean ± SD. A one-way ANOVA with *post hoc* Bonferroni was employed in the statistical analysis. **P<0.01.

We also induced a mouse model of pulmonary vascular remodeling using chronic hypoxia combined with SU5416 to disclose the probable meaning of SO_2_ in non-IgE-stimulated MC degranulation pathological model *in vivo* (**Figure 5A**). A decrease in SO_2_ content and AAT activity, along with an increase in the number of degranulated mast cells were found in lung tissues of SuHx mice compare with those of normoxic mice (**Figures 5B–D**). Simultaneously, HE and EVG staining results indicated that compared with the normoxia group, the mice in the SuHx group exhibited an increased media thickness and area of pulmonary arterioles (**Figures 5E, F**). Furthermore, after exogenous supplementation of SO_2_, the number of degranulated mast cells, the media thickness and area of the arterial were significantly reduced in the lung tissues of SuHx mice (**Figures 5D–F**). The above results suggested a potential association among the decreased SO_2_, the activation of MCs, and the pulmonary vascular remodeling in the hypoxic, a non-IgE stimulation, mouse pathological model.

### Sulfenylation at the 74^th^ cysteine of Gal-9 protein was required in the SO_2_-inhibited MC degranulation under different pathophysiological conditions

**Figure 6:**
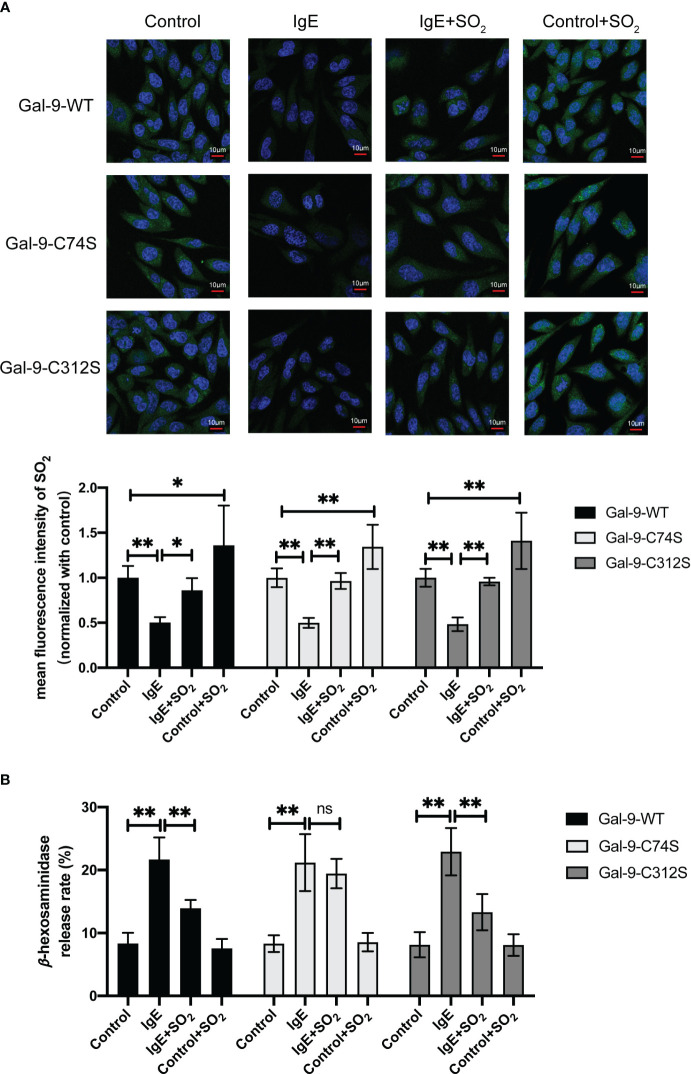
SO_2_ inhibited IgE mediated RBL-2H3 degranulation by sulfenylating the 74^th^ cysteine of Gal-9 protein. RBL-2H3 cells were transfected with Gal-9-WT, Gal-9-C74S and Gal-9-C312S plasmids. After 2 days, cells were incubated overnight with anti-DNP-IgE (100 ng/ml) and stimulated with DNP-HSA (100 ng/ml) for 1h. SO_2_ donor (100 μM) was added 1 hour before anti-DNP-IgE and DNP-HSA stimulation. **(A)** SO_2_ production in RBL-2H3 cells was tested with *in situ* fluorescent SO_2_ probe (green color, scale bar: 10 μm) (n = 9). **(B)** The release rate of β-hexosaminase in RBL-2H3 cells was determined by using colorimetric assay (n = 9). Data were expressed as mean ± SD. A one-way ANOVA with *post hoc* Bonferroni was employed in the statistical analysis. *P<0.05, **P<0.01, ns, not significant.

To demonstrate if SO_2_-mediated Gal-9 sulfenylation resulted in the inhibition of IgE-stimulated MC degranulation by SO_2_, we compared the effects of SO_2_ on the IgE-challenged RBL-2H3 cells transfected with Gal-9-WT, Gal-9-C74S mutant, and Gal-9-C312S mutant plasmids. **Figure 6A** showed that SO_2_ supplement restored the intracellular SO_2_ level in IgE-treated and increased SO_2_ content in IgE-untreated cells. In all 3 types of RBL-2H3 cells transfected with Gal-9 WT and mutant plasmids, IgE stimulation induced cell degranulation (**Figure 6B**). However, SO_2_ supplement only suppressed IgE-stimulated cell degranulation in the cells transfected with Gal-9-WT and Gal-9-C312S mutant plasmids, but not in those transfected with Gal-9-C74S mutant plasmid (**Figure 6B**). The above findings implied that SO_2_-sulfenylated Gal-9 at Cys74 was necessary to suppress IgE-activated MC degranulation.

**Figure 7:**
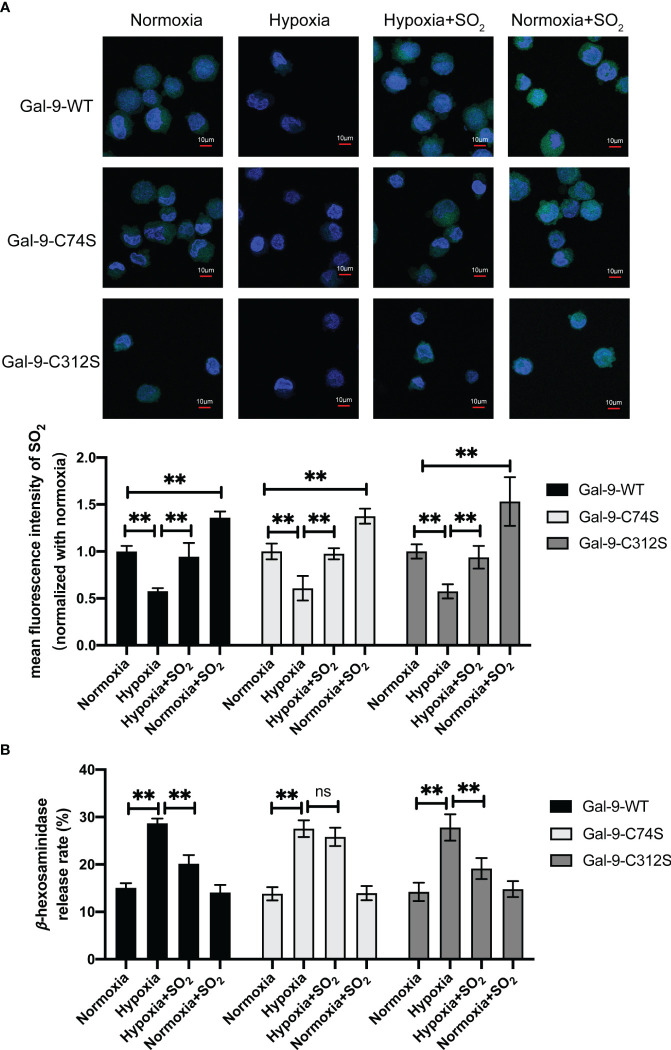
SO_2_ inhibited hypoxia-induced MC degranulation by sulfenylating the 74^th^ cysteine of Gal-9 protein. HMC-1 cells were transfected with Gal-9-WT, Gal-9-C74S and Gal-9-C312S plasmids. After 2 days, cells were then exposed to hypoxic (10%) condition for 2 days. SO_2_ donor (100 μM) was added 1 hour before hypoxic stimulation, with supplementation every 12 hours and continued until the end of the hypoxia. **(A)** SO_2_ production in HMC-1 cells was tested with *in situ* fluorescent SO_2_ probe (green color, scale bar: 10 μm) (n = 9). **(B)** The release rate of β-hexosaminase in HMC-1 cells was determined by using colorimetric assay (n = 9). Data were expressed as mean ± SD. A one-way ANOVA with *post hoc* Bonferroni was employed in the statistical analysis. **P<0.01, ns, not significant.

**Figure 8:**
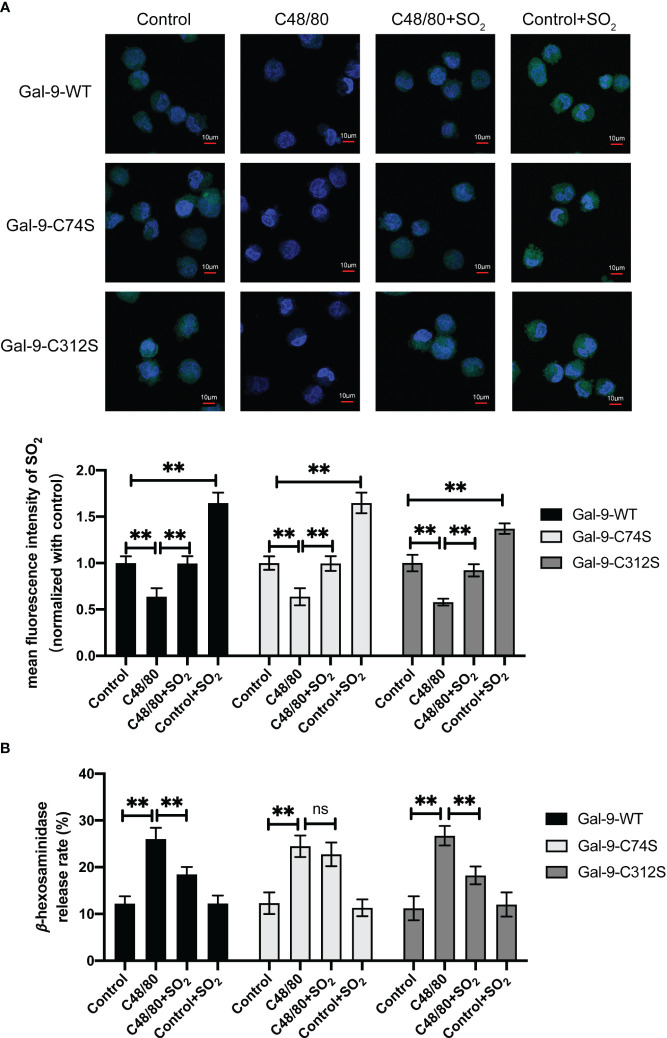
SO_2_ inhibited C48/80 induced MC degranulation by sulfenylating the 74^th^ cysteine of Gal-9 protein. HMC-1 cells were transfected with Gal-9-WT, Gal-9-C74S and Gal-9-C312S plasmids. After 2 days, cells were then sensitized with C48/80 (20 μg/ml) for 1 hour. SO_2_ donor (100 μM) was added 1 hour before C48/80 stimulation. **(A)** SO_2_ production in HMC-1 cells was tested with *in situ* fluorescent SO_2_ probe (green color, scale bar: 10 μm) (n = 9). **(B)** The release rate of β-hexosaminase in HMC-1 cells was determined by using colorimetric assay (n = 9). Data were expressed as mean ± SD. A one-way ANOVA with *post hoc* Bonferroni was employed in the statistical analysis. **P<0.01, ns, not significant.

To further clarify the significance of SO_2_-mediated Gal-9 sulfenylation in the inhibition of non-IgE-stimulated MC degranulation by SO_2_, we firstly examined the changes of AAT activity under hypoxic stimulation. The results showed that hypoxia decreased AAT activity in MC compared with normoxic cells (**Supplementary Figure 1B**). Next, we compared the effects of SO_2_ on the MCs transfected with Gal-9-WT and two mutant plasmids under hypoxic stimulation. As shown in **Figure 7A**, SO_2_ supplement increased the intracellular SO_2_ level in both hypoxic and normoxic cells. In all 3 types of HMC-1 transfected with Gal-9 WT and mutant plasmids, hypoxia induced cell degranulation (**Figure 7B**). However, SO_2_ supplement only suppressed hypoxia-induced cell degranulation in the cells transfected with Gal-9-WT and Gal-9-C312S mutant plasmids, but not in those transfected with Gal-9-C74S mutant plasmid (**Figure 7B**). Moreover, C48/80 is a compound that induces MC degranulation in a non-IgE-dependent manner (41, 42). Like the hypoxia and IgE stimulation, C48/80 also inhibited AAT activity and SO_2_ content in HMC-1 cells (**Supplementary Figure 1C**, **Figure 8A**), while SO_2_ supplement restored the intracellular SO_2_ level in the C48/80-induced cells (**Figure 8A**). Concurrently, only in the cells transfected with Gal-9-C74S mutant plasmids, SO_2_ could not prevent the C48/80-induced MC degranulation (**Figure 8B**). The above results suggested that SO_2_-sulfenylated Gal-9 at Cys74 also participated in the suppressive effect of SO_2_ on the non-IgE-stimulated pathophysiological MC degranulation.

## Discussion

In the present study, we demonstrated that SO_2_ was an important inhibitor of MC degranulation under both physiological and pathophysiological conditions. Mechanistically, sulfenylation at cysteine 74 of Gal-9 mediated the stabilization effect of SO_2_ on MC degranulation.

In the past, SO_2_ was considered an environmental pollutant and waste gas. Our research group was the first to report the existence of endogenous SO_2_/AAT pathway in the cardiovascular system (43, 44). Liu et al. have shown that SO_2_ alleviates pulmonary artery endothelial cell inflammation by inhibiting p50 nuclear translocation (45). Recent studies showed that endogenous SO_2_/AAT pathway existed in immune cells and was involved in the inflammatory regulation. For example, endogenous SO_2_ inhibits macrophage chemotaxis and inflammatory cytokine release via suppressing NF-κB pathway (46). Similarly, endogenous SO_2_/AAT pathway was reported to exist in MCs (26). In the present study, we further investigated how endogenous SO_2_ affects MC activation by transfecting AAT1 shRNA lentiviral particles into MCs. The results demonstrated that AAT1 knockdown in MCs significantly reduced both SO_2_ content and AAT activity, consequently enhancing MC degranulation. Conversely, SO_2_ donor supplementation substantially attenuated MC degranulation. These findings suggested that endogenous SO_2_ derived from AAT1 may serve as a crucial stabilizer by dampening MC degranulation under physiological conditions.

It was reported that SO_2_ induced thiol-dependent oxidative modification on specific cysteine residues to regulate protein function in the previous study (47). Therefore, a thiol-reducing agent DTT was tentatively used to estimate the possible mechanism underlying SO_2_-controlled MC degranulation. As we expected, DTT treatment blocked the SO_2_-suppressed MC degranulation, indicating that SO_2_ might regulate MC degranulation via a redox chemical modification. Furthermore, to screen the candidate target molecules mediated the SO_2_-controlled MC degranulation, a venn analysis between the SO_2_-related sulfenylome dataset and genes related to positive/negative MC regulation was conducted. The result showed that Gal-9 was the only molecule existing in the two datasets.

Gal-9 belongs to a family of β-galactoside-binding proteins involved in the regulation of cell-cell and cell-matrix interactions. Gal-9 is a lectin containing galactoside binding domain which mediates the apoptosis of Th1 cells by binding to TIM-3 (48), the apoptosis of cytotoxic T-cell following virus infection (49), and the inhibition of T cell proliferation (50) and natural killer (NK) cell function (51). In addition, Gal-9 inhibited PMA/ionomycin-mediated degranulation of HMC-1 cells by inducing ERK1/2 phosphorylation (52). The above results suggest that Gal-9 is an important regulatory molecule for controlling immune cell activity. In the present study, the results of BSA method showed that SO_2_ sulfenylated Gal-9 in HMC-1 cells and Gal-9 purified protein. Therefore, combined with the above screening results of bioinformatics analysis, we speculated that SO_2_ might inhibit mast cell degranulation by sulfenylating Gal-9.

To further locate the exact modification site of Gal-9 by SO_2_, homologous sequence analysis showed that Cys74 and Cys312 residues of the Gal-9 protein were highly conserved across 7 species. Moreover, sulfenylation at thiol group of Cys74 was found in the SO_2_-treated Gal-9 protein by LC-MS/MS analysis, which was in accordance with the screening result of SO_2_-mediated sulfenylome (30). Also, the sulfenylation of Gal-9 by SO_2_ was abolished by a mutation of Cys74 to Ser of Gal-9 but not by a mutation of Cys312, which further supported that SO_2_-induced Gal-9 sulfenylation occurs at the 74^th^ cysteine residue.

In addition to the above physiological effect, we also explored the impact of SO_2_ on mast cell degranulation under different pathophysiological stimulation such as IgE, hypoxia, and C48/80. As well known, IgE-mediated MC activation is one of the crucial mechanisms of mast cell inflammation-related disease. Activated mast cells release internal granules containing histamine, proteases, cytokines, and chemokines, thus participating in allergic responses and inflammatory processes (3, 7, 53). In the present study, we found that IgE stimulation significantly downregulated the SO_2_/AAT1 system in RBL-2H3 cells, whereas supplementation of SO_2_ recovered the IgE-suppressed SO_2_ content in MC and attenuated IgE-activated mast cell degranulation. Consistently, in a mouse PCA model, IgE/HSA stimulation resulted in a marked activation of MCs demonstrated by severe dye extravasation and tissue edema, as well as a decrease in SO_2_ content and AAT activity, which was counteracted by supplementation with SO_2_. In recent years, the MC infiltration to perivascular area and activation were found to participate in the pathogenesis of many non-allergic diseases such as cardiovascular injury diseases (54–56). Therefore, we established a mouse model of chronic hypoxia-induced mast cell activation and pulmonary vascular remodeling. As we expected, prophylactic treatment with SO_2_ donor markedly prevented MC degranulation and alleviated pulmonary artery medial remodeling. Similarly, SO_2_ donor reversed the C48/80-stimulated MC degranulation aligning with the recovery of C48/80-suppressed SO_2_ production in HMC-1 cells. Subsequently, we constructed Gal-9 WT and mutant plasmids to verify whether SO_2_ affected MC degranulation through sulfenylating Gal-9 based on the three cell models of pathological MC degranulation. The results showed that SO_2_ did not affect the IgE-, hypoxia-, or C48/80-stimulated MC degranulation in the cells overexpressing Gal-9-C74S mutant plasmid, further confirming the role of SO_2_-sulfenylated Gal-9 in the regulation of MC degranulation. Therefore, our results suggested that SO_2_ might be a key inhibitor of MC activation under IgE- and non-IgE-stimulated pathological circumstances.

There was a limitation that we employed the BALB/c or C57BL/6 mice but not mast cell specific AAT1 transgenic mice in *in vivo* experiments. Therefore, the current *in vivo* data could not demonstrate the involvement of mast cell-associated endogenous SO_2_ in the regulation of MC degranulation which needed to be clarified by further studies. Moreover, as shown in the **Supplementary Figure 2**; **Figures 6**–**8**, SO_2_ supplement could increase the intracellular SO_2_ content in the scrambled or untreated cells, but not impact MC degranulation, seemly suggesting that exogenously supplied SO_2_ donor in the normal and uninjured cells might not disrupt MC homeostasis. However, the discrepancy between the effects of exogenous and endogenous SO_2_, as well as the underlying mechanisms merit more studies to confirm.

## Conclusion

**Figure 9:**
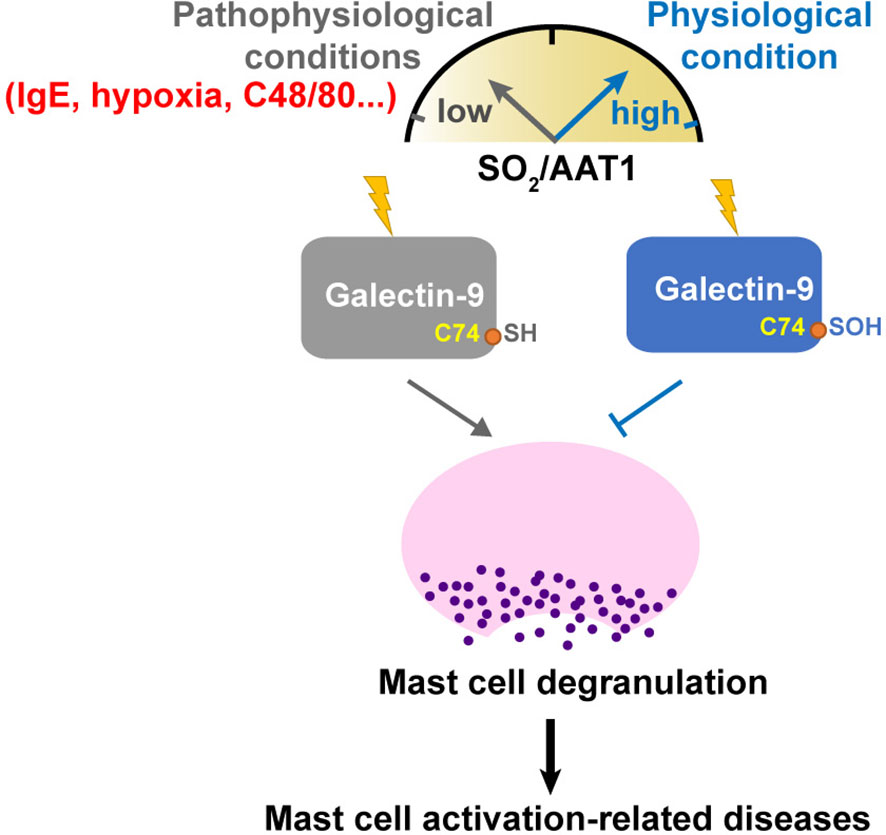
Schematic diagram illustrating a novel redox mechanism by which SO_2_ inhibits MC degranulation. SO_2_ and its sulfenylated galectin-9 at Cys74, which might be a switch of the regulation of MC degranulation and activation under physiological and pathological conditions. Downregulation of SO_2_/AAT1 pathway might be an important pathogenesis of mast cell activation-related diseases.

Collectively, our study demonstrated that endogenous SO_2_ inhibited the degranulation of MCs by sulfenylating Gal-9 at cysteine 74. The SO_2_ level in MCs and SO_2_-sulfenylated Gal-9 might be a switch of the regulation of MC degranulation and activation under physiological and pathophysiological conditions (**Figure 9**). The novel finding deepened the understanding of the mechanism underlying MC degranulation from the perspective of SO_2_-mediated redox modification. Moreover, the SO_2_/Gal-9 signal axis might serve as a new treatment avenue for MC degranulation-associated illnesses.

## Data availability statement

The datasets presented in this study can be found in online repositories. The names of the repository/repositories and accession number(s) can be found below: IPX0008057000 (iPROX).

## Ethics statement

Ethical approval was not required for the studies on humans in accordance with the local legislation and institutional requirements because only commercially available established cell lines were used. The animal study was approved by Animal Research Ethics Committee of Peking University First Hospital. The study was conducted in accordance with the local legislation and institutional requirements.

## Author contributions

JS: Formal analysis, Methodology, Visualization, Writing – original draft. JZ: Formal analysis, Methodology, Writing –original draft;. ZL: Formal analysis, Methodology, Writing – original draft. LF: Formal analysis, Methodology, Writing – original draft. JY: Formal analysis, Methodology, Writing – original draft. KL: Methodology, Writing – original draft. XY: Methodology, Writing – original draft. BL: Methodology, Writing – original draft. JD: Conceptualization, Supervision, Writing – original draft. YH: Conceptualization, Formal analysis, Supervision, Validation, Writing – original draft. HJ: Conceptualization, Formal analysis, Funding acquisition, Resources, Supervision, Validation, Writing – original draft.
