# Supplementary material for: Sulfur dioxide inhibits mast cell degranulation by sulphenylation of galectin-9 at cysteine 74
Source: Front Immunol. 2024 Jun 17;15:1369326. doi: 10.3389/fimmu.2024.1369326 (PMC11215078; doi:10.3389/fimmu.2024.1369326)
Supplement: Supplementary file 1 [file Image_1.pdf]

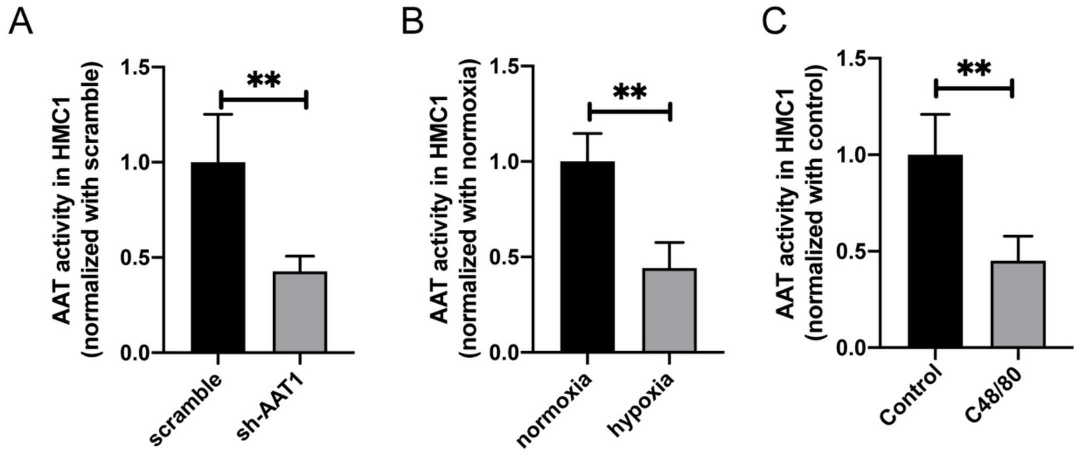

Supplementary Figure 1. Detection of AAT activity in the HMC-1 cells under physiological and pathophysiological conditions. **(A)** AAT activity in AAT1-knockdowned HMC-1 cells was detected by colorimetric assay (n = 9). **(B)** AAT activity in hypoxia-induced HMC-1 cells was detected by colorimetric assay (n = 9). **(C)** AAT activity in C48/80-stimulated HMC-1 cells was detected by colorimetric assay (n = 9). Data are expressed as mean ± SD. The two-tailed Student's *t*-test was adopted to compare two groups. $^{**}P<0.01$

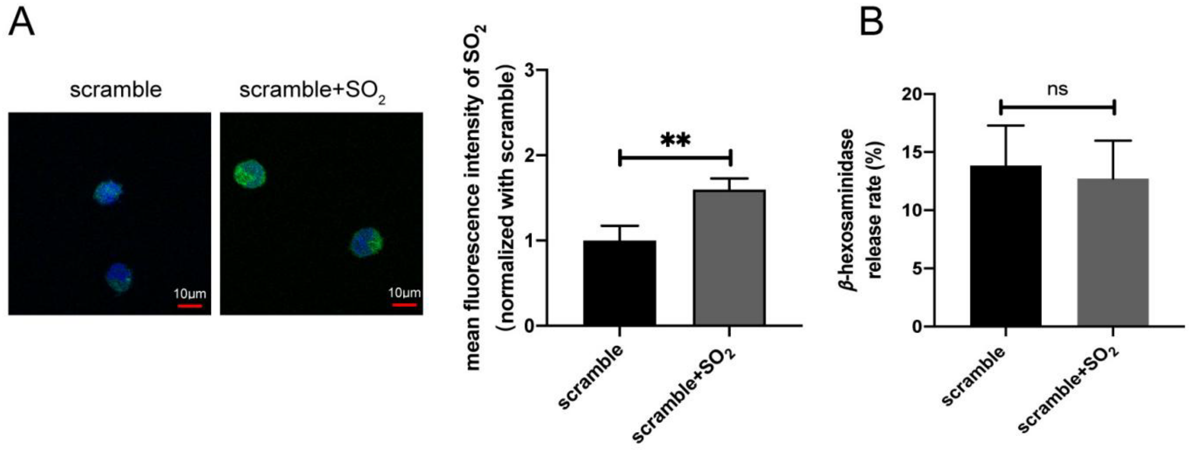

Supplementary Figure 2. The effect of SO_2 on scramble HMC-1 cells. Scramble HMC-1 cells supplemented with SO_2 donor (100 μM) for 24 hours. **(A)** SO_2 production in HMC-1 cells was tested with *in situ* fluorescent SO_2 probe (green color, scale bar: 10 μm) (n = 9). **(B)** The release rate of β-hexosaminase in HMC-1 cells was determined by using colorimetric assay (n = 9). Data are expressed as mean ± SD. The two-tailed Student's *t*-test was adopted to compare two groups. $^{**}P<0.01$, ns, not significant.
